# The iOSC3 System: Using Ontologies and SWRL Rules for Intelligent Supervision and Care of Patients with Acute Cardiac Disorders

**DOI:** 10.1155/2013/650671

**Published:** 2013-02-16

**Authors:** Marcos Martínez-Romero, José M. Vázquez-Naya, Javier Pereira, Miguel Pereira, Alejandro Pazos, Gerardo Baños

**Affiliations:** ^1^IMEDIR Center, University of A Coruña, Campus de Elviña s/n, 15071 A Coruña, Spain; ^2^Department of Information and Communication Technologies, Computer Science Faculty, University of A Coruña, 15071 A Coruña, Spain; ^3^Service of Anesthesiology, Resuscitation and Intensive Care, Meixoeiro Hospital, Meixoeiro s/n, 32600 Vigo, Spain

## Abstract

Physicians in the Intensive Care Unit (ICU) are specially trained to deal constantly with very large and complex quantities of clinical data and make quick decisions as they face complications. However, the amount of information generated and the way the data are presented may overload the cognitive skills of even experienced professionals and lead to inaccurate or erroneous actions that put patients' lives at risk. In this paper, we present the design, development, and validation of iOSC3, an ontology-based system for intelligent supervision and treatment of critical patients with acute cardiac disorders. The system analyzes the patient's condition and provides a recommendation about the treatment that should be administered to achieve the fastest possible recovery. If the recommendation is accepted by the doctor, the system automatically modifies the quantity of drugs that are being delivered to the patient. The knowledge base is constituted by an OWL ontology and a set of SWRL rules that represent the expert's knowledge. iOSC3 has been developed in collaboration with experts from the Cardiac Intensive Care Unit (CICU) of the Meixoeiro Hospital, one of the most significant hospitals in the northwest region of Spain.

## 1. Introduction

An Intensive Care Unit (ICU), sometimes called Critical Care Unit, is a special area in the hospital used to treat the most critically ill patients. ICUs are amongst the busiest and most high-pressure units in a hospital, because ICU patients require constant supervision and treatment, either because they are recovering from a major operation, have an acute illness, or have been injured in a severe accident. Medical staff in ICUs are specially trained in procedures like acute pain management and how to use specialist equipment. They also need to be able to make quick decisions in high-stress situations and stay calm, when lives are at stake.

Life of patients in the ICU depends largely on the wisdom of these decisions. Nevertheless, experts in health environments are frequently exposed to long working hours, extended days, and shift-work schedules besides high workload and psychological strain [1]. As a consequence, the human factor sometimes leads to imprecisions or mistakes in the decision making process that represent a barrier to the optimal recovery of patients. It has been reported that critically ill patients admitted to an ICU experience, on average, 1.7 medical errors each day, and numerous patients suffer a potentially life-threatening error during their stay in the ICU [2, 3]. The most frequent errors are due to the incorrect administration of medications [4, 5]. We believe that the application of Artificial Intelligence techniques to provide decision support at ICUs can help doctors to monitor and care patients, decreasing the number of treatment mistakes and improving the recovery process.

This work presents iOSC3 (intelligent Ontology-based System for Cardiac Critical Care). iOSC3 is a decision support system designed to supervise and treat patients affected by acute cardiac disorders. In hospitals with limited resources, this type of patients are treated at the general ICU. Nevertheless, when possible, they are treated at Cardiac Intensive Care Units (CICUs), which are a special type of ICU exclusively addressed to recover patients with cardiac illnesses. The system has been designed in collaboration with the CICU of the Meixoeiro Hospital (http://chuvi.sergas.es), one of the most significant hospitals in the northwest region of Spain. On the basis of the patient's vital signs at a particular time and an ontology that formalizes the expert's knowledge, the system provides a recommendation about the treatment that should be administered to achieve the fastest possible recovery.

### 1.1. Cardiac Intensive Care Units

The Cardiac Intensive Care Unit (CICU) is an area of the hospital reserved for treating patients with life-threatening cardiac medical conditions. This can include recovery from open heart surgery, heart attack recovery, or different medical issues that put the heart at risk. Medical staff in CICUs have training in specialty areas, including critical care, cardiology, and cardiac anesthesia. Their knowledge, jointly with sophisticated monitoring and support equipment, enables them to recognize problems quickly and respond effectively.

Each patient in the CICU is connected to a TV-like screen (patient monitor) which continuously acquires and displays different measures that represent body activities, such as temperature, heart rate, mean arterial pressure, and cardiac index. The patient is also connected to one or several infusion pumps, containing the drugs used to stabilize him/her. The drugs used at the CICU must be supplied judiciously and with a goal-directed approach. They are classified into two groups: vasodilators and amines (vasopressors). On the one hand, vasodilators, as the name implies, relax the smooth muscle in blood vessels, which causes the vessels to dilate and decreases blood pressure. Examples of vasodilator drugs commonly used at the CICU are nitroglycerin and nitroprusside. On the other hand, amines as dobutamine, noradrenaline, adrenaline, and dopamine have vasoconstriction properties. They increase blood pressure, which increases organ perfusion pressure and preserves distribution of cardiac output to the organs. They also improve cardiac output and oxygen delivery by decreasing the compliance of the venous compartment and thus augmenting venous return.

According to the patient's condition, CICU physicians adjust the infusion rate of vasodilators and amines in order to stabilize him/her. Sometimes, they have to integrate several rapidly changing physiologic parameters into a clear and qualitative mental image of a patient's current state and take a decision about the amount of the drugs to be administered in a short period of time. As previously explained, the system proposed in this paper is aimed to provide support to physicians in these complex decisions. The system has been developed in collaboration with experts from the CICU of the Meixoeiro Hospital (Vigo, Spain), and it has been tested in the CICU of such hospital, which is a 10-bed unit designed, equipped, and staffed to provide interdisciplinary critical care to those patients with cardiac disorders (e.g., myocardial infarction, acute coronary syndrome, congestive heart failure, pre- and postcardiac transplants, high-risk cardiac arrhythmias, etc.).

### 1.2. Ontologies in Biomedical Research

Towards the end of the 20th and beginning of the 21st centuries, and especially since the Semantic Web was conceived [6], the term “ontology” (or ontologies) gained usage in Computer Science to refer to a research area in the subfield of Artificial Intelligence mainly concerned with the semantics of concepts and with expressive processes in computer-based communications. In Computer Science, ontologies are a technique used to represent and share knowledge about a domain by modeling the things in that domain and the relationships between those things [7]. Ontologies are represented using standard, machine-processable languages (e.g., RDF [8] and OWL [9]), and they are mainly used for communication between people and organizations by providing a unified terminology that allows to reach a common level of understanding or comprehension within a particular domain.

In the biomedical field, ontologies have increasingly become an established method to represent and communicate the huge amount of knowledge about genes, diseases, biomedical processes, and so forth that has been generated during the last years [10]. Biomedical ontologies are considered crucial pieces in the development of informatics applications in several areas, such as knowledge-based decision support, terminology management, and systems interoperability and integration [11]. As a consequence, multiple biomedical ontologies have been developed and maintained, which are stored in large-scale ontology repositories available for researchers. The most popular repository of biomedical ontologies is the NCBO's BioPortal [12], a web-based, open resource that contains more than 300 ontologies with knowledge related to different biomedical topics (anatomy, gene products, immunology, phenotype, etc.) in different organisms (human, plant, mouse, microbe, etc.). 

In the medical domain, ontologies are key to reuse the large amount of complex information that is involved in many health care activities. They are used to build systems for purposes such as data annotation, information retrieval, and natural-language processing, but they are particularly useful to build knowledge-based systems that provide decision support in health care. This type of systems are generally dependent on large volumes of domain knowledge, which is extremely expensive and difficult to capture and formalize [13]. By means of ontologies, this knowledge can be represented in an application independent manner; so, it can be reused in new systems without additional knowledge extraction and development effort.

In this work, we have constructed an ontology (called Critical Cardiac Care Ontology, or C3O) that contains the knowledge used by CICU physicians to diagnose and treat patients. This ontology, which is presented in Section 3, is used by the iOSC3 system to analyze the patient's condition and to provide decision support about the treatment that should be administered to recover him/her.

### 1.3. Related Work

First contributions in the field of expert systems were made by the Artificial Intelligence community in the late 50s and early 60s, when several programs aimed at general problem solving were written. However, the first generation of clinical decision support approaches date back to the early 1970s. Popular examples are AAPHelp [14], designed to support the diagnosis of acute abdominal pain, INTERNIST-I [15], a rule-based expert system aimed to provide diagnostic support in the domain of internal medicine, and MYCIN [16], a very powerful system for diagnosing blood infections and recommending their antibiotic therapies, which has been described as the first convincing demonstration of the power of the rule-based approach in the development of robust clinical decision support systems [17]. Since the 70s, several expert systems have been proposed to solve diverse problems in medical domains, including intensive care environments. Some recent examples are an expert system for electroencephalogram monitoring in the pediatric ICU [18], an ontology-driven medical diagnosis system [19], a fuzzy logic system to regulate mean arterial pressure [20], an expert system for detection of breast cancer [1], a rule-based solution that applies semantic web techniques to ensure patient safety during breast cancer surgery [21], a system for improving specificity of alarms in critical care environments [22], a hybrid approach using case-based reasoning and rule-based reasoning for decision support in ICUs [17], and a recommendation system for antidiabetic drugs selection [23].

Table 1 summarizes the main features of iOSC3 and recent related experts systems. The systems have been classified according to the categories proposed in [24]. Despite previous work, to the best of our knowledge, no expert system has been developed to monitor and control patients at CICUs. In addition, Table 1 shows that many of the systems developed so far use traditional knowledge representation techniques that are not adequate for sharing the expert's knowledge with other professionals and to reuse it in other similar systems.

With respect to existing ontologies for the intensive care domain, during the last decade, some researchers have worked to structure and standardize existing knowledge by means of ontologies (e.g., [25, 26]). However, despite previous efforts, there is no ontology addressed to cover the cardiac intensive care domain. With this in mind, the expert system we present in this paper and the ontology that provides the underlying expert's knowledge constitute an innovate contribution in the field of medical expert systems.

## 2. The iOSC3 System

The system has been developed in Java technology by means of the Eclipse IDE and following the Unified Software Development Process [27]. The system architecture and workflow will be described in the sections which follow.

### 2.1. System Architecture

**Figure 1:**
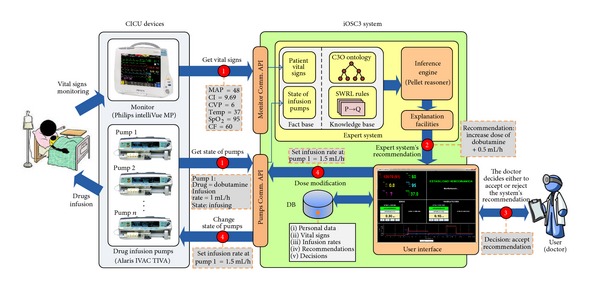
iOSC3 architecture and workflow.

The overall architecture of iOSC3 is shown in Figure 1. Each constituent will now be described in further detail.

#### 2.1.1. CICU Devices

Patients in CICUs are connected to a monitor, which is a TV-like screen that continuously acquires and displays different measures that represent body activities, such as heart rate, mean arterial pressure, and cardiac index. They are also connected to one or several infusion pumps, depending on their medical condition. These pumps are specialty devices designed to deliver controlled doses of medications that are used when it is otherwise impossible to treat a patient at the prescribed times and quantities, such as in minute quantities smaller than what a drip system can deliver. In the CICU of the Meixoeiro Hospital, the specific models of devices that are being used are Philips IntelliVue MP Series monitors and Alaris TIVA infusion pumps.

#### 2.1.2. Communication APIs

In order to enable the system's interaction with the CICU devices, it has been necessary to develop two communication APIs: the Monitor Communication API, which allows the system to establish a communication with Philips IntelliVue MP Series monitors to obtain the values of the patient's vital signs, and the Pumps Communication API, which makes it possible to obtain the state of the Alaris IVAC infusion pumps (current infusion rate and infusion state) and also to change it.

Philips IntelliVue patient monitors use a well-established communication protocol called Data Export Protocol, which is a connection-oriented, message-based request/response protocol that works on top of the standard UDP/IP transport protocol [28]. By means of the Data Export Protocol, data from the monitor can be transferred via the Local Area Network (LAN) Interface or Medical Information Bus (MIB/RS232) Interface to an external computer.

We have developed an API (Monitor Communication API) that implements the Data Export Protocol in order to obtain data from the IntelliVue monitor via the LAN interface. The iOSC3 system is connected to the IntelliVue monitor using a standard unshielded LAN cable with an RJ45 connector. The network IP address is automatically configured with the standard Bootstrap Protocol (BootP).

The communication with the Alaris syringe pumps is achieved by means of the Alaris Pump Communications Protocol [29]. This protocol is used for the pump models GS, GH, CC, and TIVA with software version V1.5.10 (or V1.6.2 for TIVA models) and above. The communications model is a point-to-point connection between two communicating parties (a client and a server), and it has been designed to support two types of connection: conventional RS232 serial interface and IrDA infrared specification. 

Our Pumps Communication API allows to communicate the system with the Alaris pumps by means of the RS232 serial interface. It enables the system to obtain the state of the infusion pump, as well as to modify it.

#### 2.1.3. Expert System

The decision making process provided by iOSC3 is supported by a specialized expert system that is in charge of analyzing the patient's condition and providing a recommendation about the treatment that should be applied to stabilize him/her. The expert system has 4 essential components: knowledge base, fact base, inference engine, and explanation facilities.

- The *knowledge base* contains all of the relevant domain-specific information, permitting the system to act as a specialized problem solver for the CICU domain. It has been constructed in direct collaboration with the physicians at the CICU of the Meixoeiro Hospital, who are the experts in the problem domain. It consists of two essential components: an ontology, which has been written in a formal language and represents the concepts used at the CICU (e.g., vital sign, medical device, infusion pump, drug, etc.) and their relations (e.g., cardiac index “is a” vital sign) and a group of IF-THEN rules that represent the protocol used by the CICU doctors to diagnose and treat patients. A detailed explanation about how the ontology and the rules were built will be provided in Section 3.
- The *fact base* contains a collection of facts against which rule conditions are evaluated. It contains the values of the patient's vital signs and the state of each infusion pump in a given time. These facts match the left sides of production rules to determine eligible rules for firing. As previously explained, these data are obtained from the CICU patient monitor and the infusion pumps by means of the Monitor and Pumps Communication APIs that have been developed.
- The *inference engine* is the core of the expert system. It links the rules given in the knowledge base with the fact base and performs reasoning to reach a solution. As inference engine, we have decided to use Pellet [30], which is the leading choice for applications that need to reason about knowledge represented using OWL ontologies [9] and SWRL rules [31].
- The *explanation facilities* enable the user to ask the expert system how a particular conclusion is reached. It has been shown that physicians are more likely to adhere to expert system recommendations when quality explanation facilities are available [32]. In fact, it has been reported that expert system advice is usually ignored when it is not accompanied by an explanation, even when users acknowledge its global good performance [33]. The iOSC3 system uses the standard explanation facilities provided by Pellet, which include explanation for SWRL rules.

The knowledge base and the facts base are loaded by the inference engine by means of Jena (http://jena.apache.org/), which is a Java framework for building Semantic Web applications that provides a complete collection of tools to manage ontologies. On the basis of this knowledge, the inference engine executes the reasoning process and provides the doctor with a recommendation about the patient's treatment (see step 2 in Figure 1).

#### 2.1.4. Graphical User Interface

The user interface enables the communication between the doctor and the expert system. It has two main windows: the case management window, which allows the doctor to store and query the patient's general information, and the intelligent care window, which is the interface that the doctor uses to supervise and treat the patient.

**Figure 2:**
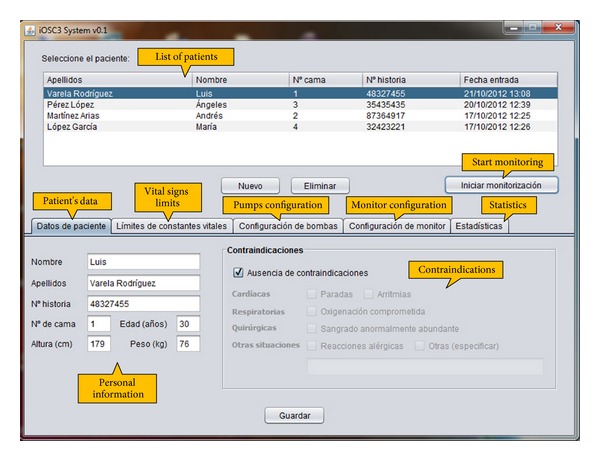
Case management window, showing the content of the patient's data tab.

The case management window (see Figure 2) allows the user to store and query information about the patients that are going to be treated by the system and configure the CICU devices for each one of them. This window has the following tabs.

**Figure 3:**
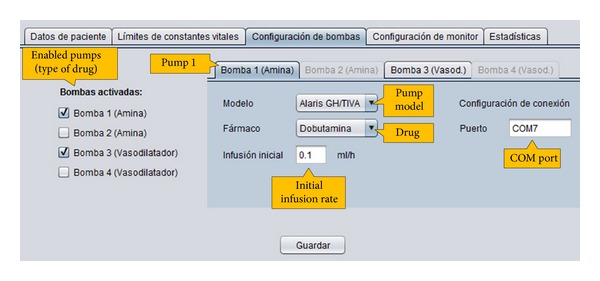
Pumps configuration tab.

- *Patient's Data.* The content of this tab is shown in Figure 2. It is aimed to save the patient's name, surname, clinical history number, bed number, age, height, and weight. It also allows the doctor to point out if the patient has some contraindications (cardiac, respiratory, surgical, or others) that may require different medical attention and may prevent the use of the system.
- *Vital Signs Limits.* This tab allows the doctor to input the top and bottom limits for the patient's vital signs (mean arterial pressure, cardiac frequency, cardiac index, central venous pressure, temperature, and O2 saturation). These limits will be used by the expert system to determine the stability or instability of the patient and suggest appropriate treatment to ensure their recovery. As an example, the top and bottom limits for the mean arterial pressure could be 50 mmHg and 130 mmHg, respectively.
- *Pumps Configuration.* The content of this tab is shown in Figure 3. It allows to set the number of pumps that are connected to the patient, as well as the specific type of drug in each pump and the initial infusion rate. The user also has to input the COM port number to which the pump is connected. As previously explained, current version of iOSC3 only allows the communication with infusion pumps that use the Alaris Pump Communications Protocol. However, due to its modular architecture, the system can be easily modified to allow the communication with other pump models. Regarding the number of pumps, the system allows to work with 2 pumps with amine drug and 2 pumps with vasodilator drug as maximum. This number of pumps, filled with the appropriate drugs, is enough to solve all the possible situations.
- *Monitor Configuration.* The aim of this tab is to configure the connection to the patient monitor. Communication is achieved through LAN connection; so, it is necessary to provide the monitor IP (e.g., 169.254.127.255) and the communication port (e.g., 24105). Current version of the system is prepared to interact with Philips IntelliVue MP Series monitors.
- *Statistics.* This tab is used to show different statistics about the patient's treatment that can be useful to extract conclusions about the usefulness of the system. Examples of these statistics are patient's recovery time, total amount of drug supplied to each patient, and number of system's recommendations accepted and refused by the doctor.

**Figure 4:**
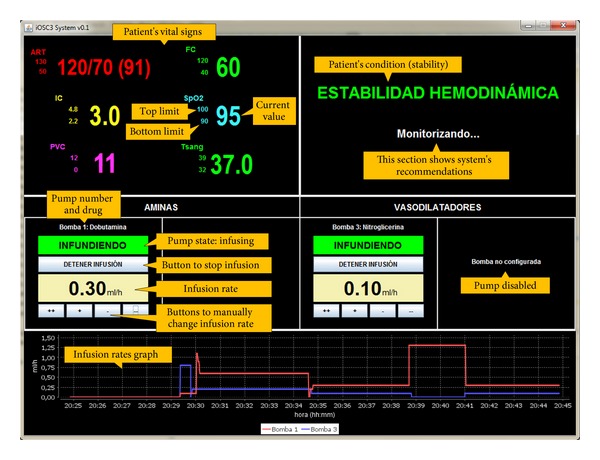
Intelligent care window.

When the user has entered all the data for a specific medical case, he/she can start the execution of the intelligent decision support system by clicking the “Start monitoring” button. Then, the intelligent care window is opened (see Figure 4). This window allows the doctor to monitor the patient's condition and supervise the treatment that is being supplied. It is divided into four sections, which display the following information.

- *Patient's Vital Signs.* The current values of the patient's vital signs, as well as the top and bottom limits that have been set for each parameter.
- *State of Infusion Pumps.* The current state of the infusion pumps that are connected to the patient. For each pump, the screen shows the pump name and drug (e.g., name: pump1; drug: dobutamine), the infusion state (i.e., infusing or stopped), and the current infusion rate (e.g., 1.2 mL/h). It also provides buttons to start or stop the infusion and to modify the infusion rate.
- *Evolution of Infusion Rates.* This region displays a graph that represents the infusion rate applied at each infusion pump during a certain period of time (e.g., last 20 minutes).
- *Treatment Recommendation.* The expert system analyzes the patient's condition and the treatment that is being administered, and if the patient is in a situation of instability according to the expert knowledge contained in the knowledge base, the system provides a recommendation about the modifications that should be done on the infusion pumps to stabilize him/her. The doctor can accept or reject the system's recommendation. If the recommendation is accepted, the system automatically communicates with the infusion pumps to achieve the necessary modifications. The physician also can edit the infusion rate suggested by the system and set the infusion rate that he/she considers appropriate. If the doctor refuses the recommendation, the treatment is not modified and the system continues monitoring the patient.

#### 2.1.5. Database

All the data managed by the iOSC3 system are stored into a MySQL database for subsequent analysis. For each patient, the system stores personal information, contraindications, limits of vital signs, pumps, and monitor configuration. In addition, while the patient is being monitored, the system periodically (each 5 seconds) stores his/her vital sign values, the state and infusion rates at each pump, the system's recommendations, and the decisions taken by the doctor. All these data are essential to extract conclusions about the usefulness of the system and to identify the aspects that should be improved.

**Figure 5:**
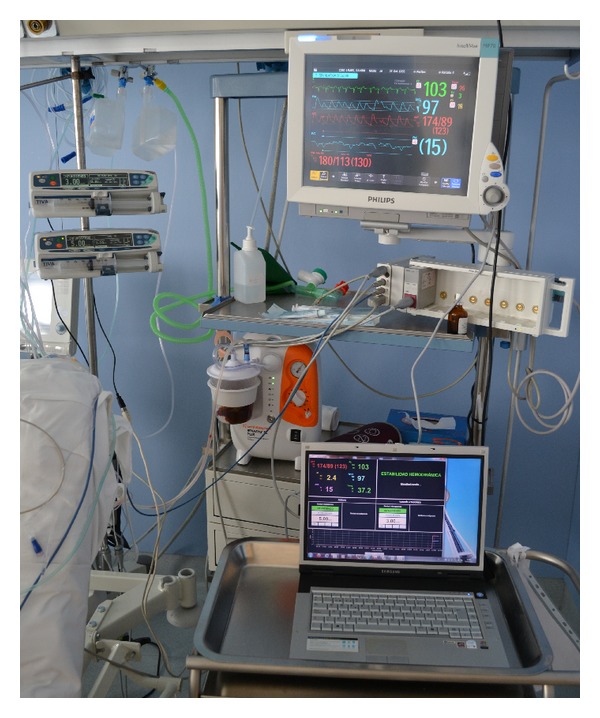
iOSC3 installed in the CICU of the Meixoeiro Hospital.

Figure 5 shows the system installed in the CICU of the Meixoeiro Hospital. It is possible to see 2 Alaris infusion pumps, a Philips IntelliVue MP70 monitor, and a laptop that shows the system's intelligent care window.

## 3. Building the Critical Cardiac Care Ontology (C3O)

**Figure 6:**
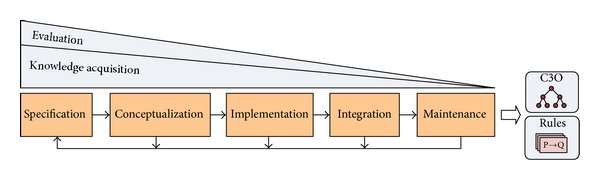
Ontology development process (a simplified version of the Methontology lifecycle [46]).

At this section, we explain the methods followed to extract the expert's knowledge and represent it formally as an OWL ontology. The ontology building process was guided by Methontology [34], one of the most popular methodologies for ontology development, following a bottom-up approach and based on the OBO Foundry principles (http://www.obofoundry.org/crit.shtml). The ontology development process was achieved according to the activities shown in Figure 6, which are described later.

### 3.1. Specification

The goal of this stage was to produce an informal ontology specification document written in natural language, that described general aspects of the ontology and its intended use. We wrote a document describing the ontology domain, purpose of the ontology, scenarios of use, intended users, level of formality, list of terms to be represented, and related bibliographic references. Initially, this document reflected many aspects that were not clear, but it has been gradually refined and expanded throughout the rest of the development process, giving rise to a structured text that reflects the ontology requirements in a concise and clear manner.

### 3.2. Knowledge Acquisition

This phase occurs largely in parallel with the rest of the ontology development process, especially with the specification stage, and decreases as the process moves forward. At this stage, we held a variety of meetings and interviews with the CICU doctors, who were asked to describe in detail the procedures they employ to monitor and treat patients. These interviews allowed to acquire a wide set of documented information (interview transcripts, books, scientific papers, diagrams, technical manuals, etc.) about the protocols followed in the CICU to treat patients, as well as regarding the technical details of the medical devices used at the CICU (patient monitor and drug infusion pumps).

### 3.3. Conceptualization

**Figure 7:**
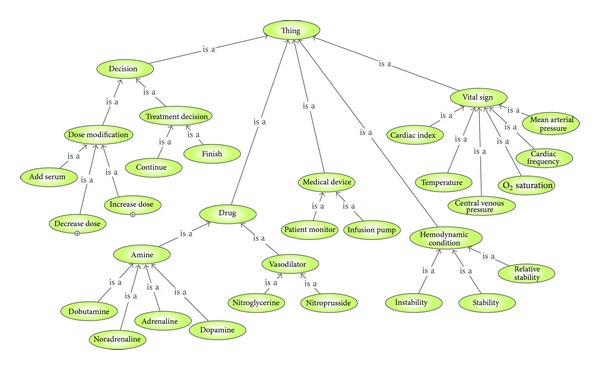
Fragment of the C3O hierarchy of classes.

At this point, we structured the domain knowledge in a conceptual model. We identified the key terms (e.g., cardiac frequency, infusion pump, drug, etc.) and the relations between those terms (e.g., nitroglycerin “is a” vasodilator agent) and organized them in a taxonomic “is a” hierarchy following a top-bottom approach (see Figure 7). We also extracted the main rules that guide the decision making process and structured them as a list of IF-THEN rules.  Table 2 provides a description of the most relevant classes and properties.

### 3.4. Implementation

**Figure 8:**
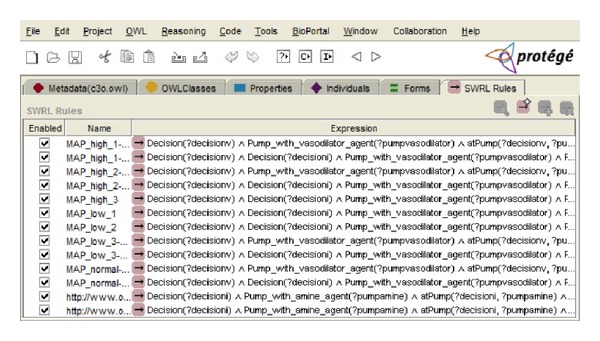
Screenshot of the SWRL Editor showing some of the C3O rules.

This activity involved the representation of the ontology in a formal language. The ontology was formalized in OWL DL, a description logics-based sublanguage of the Ontology Web Language (OWL) [35]. It was chosen because it is highly expressive and it still retains computational completeness and decidability. In addition, several well-known reasoning systems are available for OWL DL, such as Pellet. The ontology was built using the Stanford University ontology editor Protégé (version 3.4.7) [36]. The inference rules were written in the Semantic Web Rule Language (SWRL) [31], which is the rule representation language recommended by the Semantic Web community and allows to express rules on the basis of ontology concepts. The rules were written using the SWRL Editor (see Figure 8), a development environment for working with SWRL rules in Protégé-OWL. When editing rules in this environment, users can directly refer to OWL classes, properties, and individuals within an OWL ontology. They also have direct access to a full set of built-ins described in the SWRL built-in specification and to all of the XML Schema data types [37]. The rules were stored as OWL individuals in the C3O ontology.  Box 1 shows an example of rule used by the expert system written both in natural language and in the Semantic Web Rule Language.

The resulting ontology has been called Cardiac Critical Care Ontology (C3O). It contains 40 well-defined terms (classes) frequently used by experts in the area of CICUs organized as a taxonomy, 1 object property, 5 datatype properties, and a set of inference rules that guide the decision making process. The C3O ontology in OWL format is publicly available at http://tinyurl.com/cyeqq6x. 

### 3.5. Integration

According to the knowledge-reuse principles proposed by the OBO Foundry [38], at this phase, we incorporated to the C3O ontology knowledge already provided by other ontologies. We checked if the identified concepts were already contained in other existing biomedical ontologies. Carrying out this process manually is a hard and time-consuming task; so, we used a biomedical ontology selection tool (the BIOSS system (http://bioss.ontologyselection.com/) [39, 40]). We observed that most of the concepts were distributed across different ontologies. Also, some concepts had not been previously defined. As an example, the MeSH ontology (version 2009_02_13) contains the concepts “dobutamine” and “infusion pump,” but it does not contain the concept “Mean Arterial Pressure,” which is contained in the NCI Thesaurus ontology (version 2008_05D). We referenced the concepts contained in other ontologies and created the concepts that had not been previously defined.

### 3.6. Evaluation

This task has been achieved during each phase and between phases. The term evaluation subsumes the terms verification and validation. As explained by Fernandez et al., verification refers to the technical process that guarantees the correctness of an ontology with respect to a frame of reference. Validation guarantees that the ontology corresponds to the system that it is supposed to represent [41]. We reviewed the ontology and the set of rules with respect to the requirements specification document in order to detect and solve incompleteness, inconsistencies, and redundancies.

### 3.7. Maintenance

Ontologies applied in real-world settings are continuously evolving [42]. When the requirements change, it is possible that some ontological knowledge is no more relevant to the application domain. In that case, this knowledge has to be removed from the ontology. In a similar way, sometimes it is necessary to add to the ontology new terms or relations that are necessary to support new application requirements.

## 4. Evaluation and Results

Evaluation of decision support systems is a global term that comprises two main stages: evaluation of the intrinsic properties of the system (technical evaluation) and evaluation of its actual use and utility (user's evaluation or assessment). Technical evaluation is divided in two tasks: verification and validation. Verification and validation assess the consistency, correctness, and completeness of the knowledge within the expert system, the quality of the solutions provided by the system, and the ability of the system to produce the same results given the same inputs [43].

In this section, we describe how the expert system was verified and validated. User's evaluation reflects the acceptance of the system by the end users and its performance in the field, and it will be suggested as a future work. It will require to design a clinical study to test how the system affects the structure, process, and outcome of health care encounters.

Verification of an expert system refers to “building the system right,” that is, checking that the system is a correct implementation of the specification. This stage was carried out by the development team according to the requirements extracted from the medical experts. One of the major tasks in verifying expert systems is the verification of the knowledge contained within the knowledge base. This type of verification assesses the accuracy of the knowledge, because inaccuracy in the knowledge base results in inaccuracy for the whole expert system. As explained in Section 3.6, this kind of verification was achieved during the ontology development process.

Validation generally is regarded as a more complex task than verification. It refers to “building the right system,” that is, substantiating that it performs with an acceptable level of accuracy the real-world tasks for which it was intended. Validation is the cornerstone of evaluation since, for example, a highly efficient implementation of an invalid system is useless [44]. There are different ways to validate an expert system. We have decided to achieve a validation based on test cases, because this method has been reported to be the dominant strategy for the systemic validation of expert systems [45]. 

Preparing a high-quality set of test cases is crucial to achieve an accurate and complete validation. This set must offer an adequate domain coverage. A good criterion to construct the set of test cases is covering all the possible system outputs that could be generated in a particular moment [19]. We defined a set of 14 domain-representative test cases. Then, we executed the system and compared the system's output with the expected output, provided by a medical expert. Each test case consisted of a set of input parameters (values of patient's vital signs and pump infusion rates) and an expected output (system's recommendation). The test cases were created on the basis of real data, collected at the CICU and inserted into the system by means of a software application. An example of one of these test cases is shown in Table 3. After executing all test cases, the system was able to achieve an overall precision of 100% (see Table 4).

In spite of that a comprehensive user's evaluation is suggested as a future work, during the development of the system, we have received some remarks from the physicians at the CICU of the Meixoeiro Hospital that may be considered of interest. The feedback received is summarized as follows.

The medical experts who have interacted with the system or discussed about it during the development process consider that it can be a useful support tool for their daily work, especially in situations of high workload or uncertainty. The system is able to provide a recommendation about the patient's treatment in a matter of a few seconds, and physicians think that, even if the system is not completely accurate, it can be extremely useful to guide them to the proper decision. Other comment that we have received is that physicians are very comfortable with the system's user interface. The interface has been designed following the look and feel (i.e., colors, screen distribution, fonts, etc.) of existing patient monitors; so, it looks familiar to the medical experts, and they learn how to use it quickly. Finally, another feature of iOSC3 that is considered very valuable by physicians is that all the information about the status of the patient and the infusion pumps is stored into a database for further analysis, regardless of whether the system is used for decision support or not. After a patient leaves the CICU, physicians can use all these data to study his/her evolution and discover human errors that have occurred, identify aspects of medical protocols that could be improved, compare the effectiveness of different drugs, and so forth.

## 5. Conclusions and Future Research

Patient monitoring and management at the ICU is a difficult task involving acquisition and processing of a huge volume of complex data. ICU physicians have to analyze and interpret these data to make quick decisions, which frequently imply modifications on the dosage of drugs being administered. However, the human factor is sometimes a source of mistakes that lead to inaccurate or erroneous decisions about patients' care. The development of decision support solutions that help physicians to manage and process the continuous flow of information and to make quick and reliable decisions about the treatment of patients can provide multiple benefits, including improved quality of care and an overall reduction in cost. In general, experts in critical environments are very interested in the development of such kind of systems because they make their daily work easier and help them to avoid mistakes in patients' treatment.

In this work, an ontology-based system for intelligent supervision and treatment of critical patients with acute cardiac disorders has been presented. The system is based on expert knowledge, which has been formalized in the form of an ontology and a set of semantic rules. This ontology contains the main concepts used by experts in CICUs, the relationships between these concepts, and the set of inference rules that guide the decision making process. To the best of our knowledge, this is the first time that knowledge to treat critical cardiac patients has been formally represented as an ontology and used as the basis to build a decision support system.

Future research is mainly focused on designing a study to be achieved at the CICU of the Meixoeiro Hospital that will allow to evaluate the use of the system in clinical practice and how it affects the treatment and recovery of patients.

## Figures and Tables

**Box 1:**
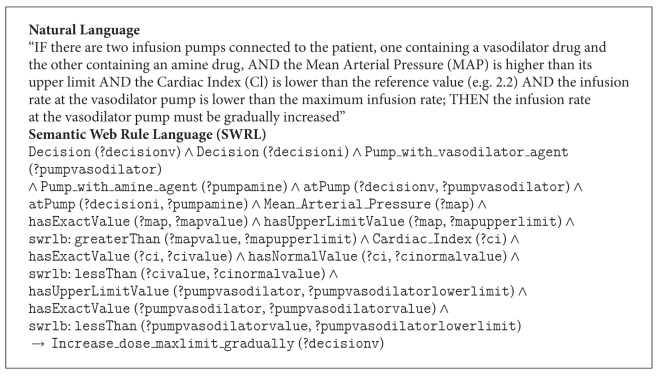
Example of rule written in natural language and SWRL.

**Table 1:** Comparison of iOSC3 with related medical expert systems. Each system is classified according to the categories proposed in [24]. The last column summarizes the main techniques or technologies used for knowledge representation and processing.

System	Category	Application	Techniques/technologies
iOSC3	Ontology-based, rule-based	Decision support in cardiac ICUs	OWL ontology, SWRL rules, Pellet reasoner
Chen et al. (2012) [23]	Ontology-based, rule-based	Antidiabetic drugs selection	OWL ontology, SWRL rules, JESS engine
ODDIN (2010) [19]	Ontology-based, rule-based, probabilistic	Differential diagnosis in medicine	OWL ontology, Jena rules
Nocedal et al. (2010) [21]	Rule-based	Breast cancer treatment	OWL ontology, inference rules
Kumar et al. (2009) [17]	Case-based, rule-based	Clinical decision support in ICUs	Rules in XML format
Blum et al. (2009) [22]	Intelligent agents, rule-based	Improving physiologic alarms in critical care	Inference engine implemented using a stored SQL procedure
Karabatak and Ince (2009) [1]	Association rules, neural network	Breast cancer detection	Association rules for feature extraction and multilayer perceptron for intelligent classification
Si et al. (1998) [18]	Fuzzy logic, neural network	Electroencephalogram monitoring in pediatric ICUs	Statistical comparison of features, fuzzy logic for feature classification, and neural networks for EEG assessment

**Table 2:** Main classes and properties contained in the C3O ontology.

Name	Type	Description
Decision	Class	A class that represents the different judgments made at the CICU to treat a patient.
Dose_modification	Class	Adjustment of the amount of drugs (infusion rate) that is being supplied to the patient.
Treatment_decision	Class	Choice about the treatment that is being administered to the patient.
Drug	Class	Substance used at the CICU to treat patients.
Amine	Class	Drug with vasoconstriction properties. Amines increase blood pressure, which raises organ perfusion pressure and preserves distribution of cardiac output to the organs. Examples: dobutamine, noradrenaline, and adrenaline.
Vasodilator	Class	Drug that relax the smooth muscle in blood vessels, which causes the vessels to dilate and decreases blood pressure. Examples: nitroglycerine and nitroprusside.
Medical_device	Class	Equipment used at the CICU to monitor or treat patients. Examples: patient monitor and infusion pump.
Hemodynamic_condition	Class	State of health of the patient with respect to the situation of the forces the heart has to develop to circulate blood through the cardiovascular system.
Vital_sign	Class	Indicator of a patient's general physical condition. Examples: cardiac index, temperature, and mean arterial pressure.
atPump	Object property	A property which allows to represent the dose modification that has to be applied at a specific infusion pump (domain: dose_modification; range: infusion_pump).
hasValue	Datatype property	A property which represents the values and limits of the patient's vital signs by means of its children: hasExactValue, hasLowerLimitValue, hasNormalValue, and hasUpperLimitValue.

**Table 3:** Example of test case. The MAP value (40.0) is lower than its lower limit (50.0). Other patient parameters have normal values. In this situation, the decision would be to decrease the vasodilator infusion rate and increase the amine infusion rate.

	Parameter	Unit	Value	Lower limit	Upper limit
Vital parameters	Mean arterial pressure (MAP)	mmHg	40.0	50.0	90.0
	Oxygen saturation (SpO_2_)	%	92.0	90.0	100.0
	Central venous pressure (CVP)	mmHg	10.0	4.0	20.0
	Cardiac frequency (CF)	bpm	76.0	40.0	120.0
	Cardiac index (CI)	L/min/m^2^	2.3	Ref. value: 2.2	
	Temp (T)	°C	37.1	32.0	39.0

Infusion rates	Vasodilator pump	mL/h	4.0	0.0	10.0
	Amine pump	mL/h	2.0	0.0	10.0

Expected recommendation	Decrease the infusion rate at the vasodilator pump and increase the infusion rate at the amine pump				
	until the MAP reaches normal values				

**Table 4:** Summary of validation results.

Parameter	Value
Number of test cases	14
Correct decisions	14
Incorrect decisions	0
Precision	100%
